# Modelling of Longitudinal Elastic Wave Propagation in a Steel Rod Using the Discrete Element Method

**DOI:** 10.3390/ma15082738

**Published:** 2022-04-08

**Authors:** Magdalena Knak, Michał Nitka, Erwin Wojtczak, Magdalena Rucka

**Affiliations:** 1 Department of Mechanics of Materials and Structures, Faculty of Civil and Environmental Engineering, Gdansk University of Technology, Narutowicza 11/12, 80-233 Gdansk, Poland; erwin.wojtczak@pg.edu.pl (E.W.); magdalena.rucka@pg.edu.pl (M.R.); 2 Department of Buildings Structures and Material Engineering, Faculty of Civil and Environmental Engineering, Gdansk University of Technology, Narutowicza 11/12, 80-233 Gdansk, Poland; michal.nitka@pg.edu.pl

**Keywords:** guided waves, longitudinal wave, discrete element method, finite element method, numerical modelling, dispersion curves

## Abstract

The paper deals with the issue of modelling elastic wave propagation using the discrete element method (DEM). The case of a longitudinal wave in a rod with a circular cross-section was considered. A novel, complex algorithm consisting of the preparation of models and simulation of elastic waves was developed. A series of DEM models were prepared for simulations, differing in discretisation and material parameters. Additional calculations with the finite element method (FEM) were performed. Numerical wave signals were obtained from each simulation and compared with experimental results to choose the best DEM model based on the correlation between the waveforms. Moreover, dispersion curves were prepared for each model to verify the agreement with the Pochhammer-Chree wave propagation theory. Both experimental and theoretical approaches indicated the same model as the most suitable. The analysis results allowed stating that DEM can be successfully used for modelling wave propagation in structural rods.

## 1. Introduction

Ultrasonic guided waves are widely used in engineering structures’ non-destructive testing (NDT). They are mostly applied to detect and localise damage [1,2,3] or determine elastic [4,5] and thermal properties [6,7,8]. For a comprehensive interpretation of results, experimental investigations can be enhanced with wave propagation simulations in numerical models. The calculations are typically performed in software implementing the finite element method (FEM). Modelling elastic wave propagation in the FEM is widely described in structural elements like rods and beams, plates and other more complex structures [3,9,10,11]. The FEM is mostly used for modelling structures made of homogeneous materials (e.g., isotropic steel or orthotropic composites). However, it can also be successfully applied in the case of highly heterogeneous materials (e.g., concrete). Several works consider the mesoscale structure of materials in terms of wave propagation [12,13]. For modelling and characterising the fracture properties of concrete, the discrete element method (DEM) is increasingly used, which allows for the study of its mechanical behaviour at the aggregate level. However, for discrete element-based techniques, the problem of wave propagation is not widely considered.

The discrete element method is a numerical modelling approach with many applications in various industries, e.g., hydromechanics, grinding, or even medicine [14,15,16]. The modelled structure is built from particles (mostly discs and spheres) that may interact with each other. For this reason, it is very popular in the fracture analysis of granular media such as sand [14,15,16,17] or concrete elements [18,19,20,21,22,23,24,25,26]. The geometry of the model, such as the interactions between particles, are crucial issues. The DEM allows computing the motion of a large number of elements. Its main advantage is the high level of detail of the behaviour of each particle (at the macro- or even micro-level). The particles are considered perfectly rigid bodies but with smooth (soft) contacts (so-called overlaps). The DEM is based on the use of an explicit numerical integration scheme where the interaction of particles is monitored contact by contact. The motion of the particles is modelled one by one. It is based on Newton’s second law, which is discretised by a finite difference shape, solved explicitly. In general, the DEM can be considered more complex than FEM, thus enabling an accurate reconstruction of the actual concrete mesostructure and a realistic prediction of fracture. An emerging research area is the investigation of the scattering of elastic waves within heterogeneous materials. However, the literature dealing with wave propagation problems in the DEM is still limited. An example of this kind of research was presented by Rojek et al. [27]. The authors described a micro-macro relationship in wave propagation simulation using the DEM. Their work involved calculations performed on 2D models using DEMPack software. The presented results confirmed the possibility of application of DEM for the simulation of guided wave propagation in solid materials.

The discrete element method is mainly used for modelling heterogeneous media. However, since concrete structures usually are reinforced by bars, reinforcement cannot be ignored in modelling, even though it is mostly made of steel. Thus, the current work is focused on the steel rod problem as the first step for further analysis directed to wave propagation-based diagnostics of reinforced concrete structures. The paper presents the guided wave propagation problem formulation in a circular rod using the DEM. A novel model preparation and calibration algorithm based on experimental and FEM analysis is developed. The main attention is paid to the process of the determination of discretisation and mechanical parameters.

The paper is structured as follows. The theoretical background of the DEM is given in Section 2, including basic formulae and computational methods. The proposed methodology for elastic wave modelling using the DEM is shown in Section 3. The description of the tested object with the details of experimental investigations and numerical calculations using the FEM and DEM methods is presented in Section 4. Moving on, Section 5 shows the results of the analyses performed. The guided waveforms obtained with the DEM were correlated with the experimental and FEM results. The theoretical dispersion curves were also incorporated to verify the proposed modelling algorithm. The paper completes in Section 6, which presents the main findings of the current study.

## 2. The Theoretical Background

### 2.1. Guided Waves in a Circular Rod

Longitudinal guided wave modes propagating in a circular rod with a radius *r* can be described by the Pochhammer-Chree frequency equation [28]:

(1) $$\frac{2\alpha }{r}\left(\beta^{2}+k^{2}\right)J_{1}\left(\alpha r\right)J_{1}\left(\beta r\right)-{\left(\beta^{2}-k^{2}\right)}^{2}J_{0}\left(\alpha r\right)J_{1}\left(\beta r\right)-4k^{2}\alpha \beta J_{1}\left(\alpha r\right)J_{0}\left(\beta r\right)=0,$$

where *J*_0_ and *J*_1_ stand for Bessel’s functions of the first kind, parameters *α* and *β* are related to the wavenumber *k*, the angular frequency *ω* by:

(2) $$\alpha^{2}=\frac{\omega^{2}}{c_{P}^{2}}-k^{2},\;\beta^{2}=\frac{\omega^{2}}{c_{S}^{2}}-k^{2}$$

In Equation (2), $c_{P}^{2}$ and $c_{S}^{2}$ denote the velocities of longitudinal and shear waves, respectively, and they are given by:

(3) $$c_{P}=\sqrt{\frac{E\left(1-\nu \right)}{\rho \left(1+\nu \right)\left(1-2\nu \right)}},\;c_{S}=\sqrt{\frac{E}{2\rho \left(1+\nu \right)}}$$

where *E* is the young’s modulus, ρ is the mass density and ν is the Poisson ratio. The solution of Equation (1) provides dispersion curves, which relate the group velocity to the angular frequency by the relation:

(4) $$c_{g}=\frac{d\omega }{dk}$$

### 2.2. Outline of Discrete Element Method

The numerical analysis was performed with the open-source code Yade [29,30]. The algorithm for DEM calculations using spherical elements can be as follows. First, the position of every particle is established. Then, the contact between each particle and its neighbours (adjacent particles or other objects like walls or boxes) is found. If the contact exists, the overlap *u* is calculated from the equation:

(5) $$u=d-\left(R_{A}+R_{B}\right),$$

where *d* is the distance between the centres of the elements and *R_A_* and *R_B_* are the radius of the elements in contact. Compression forces exist if the overlap is negative (if it is positive, tension appears).

**Figure 1:**
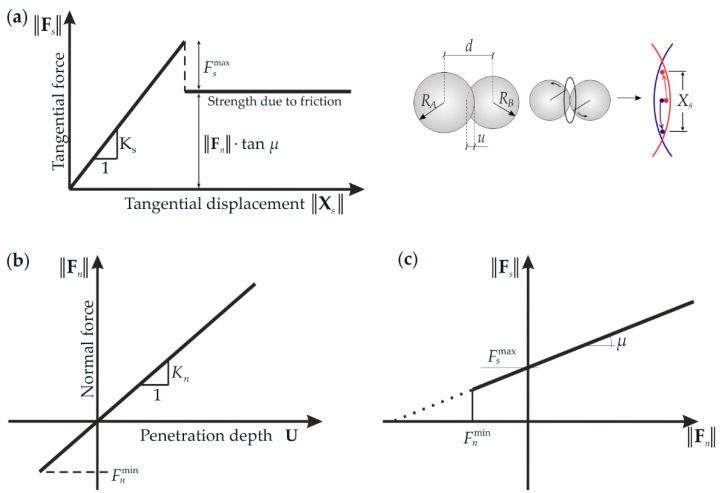
Mechanical response of DEM: (**a**) tangential contact model, (**b**) normal contact model, and (**c**) modified Mohr-Coulomb model [29].

**Figure 2:**
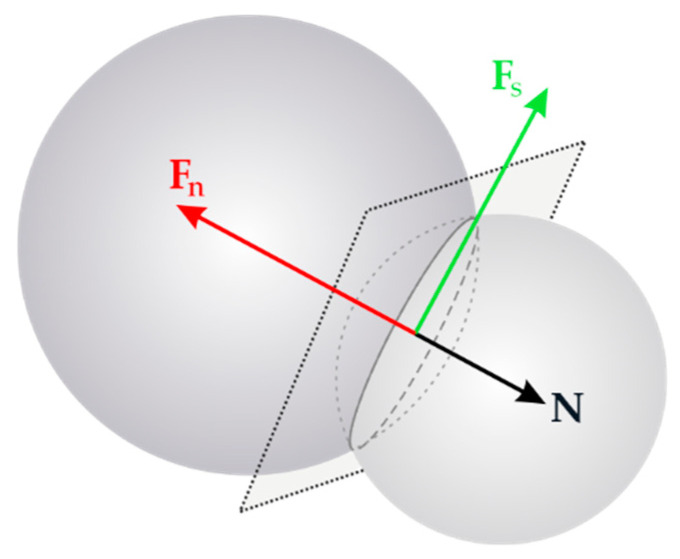
Two spheres in contact with the forces and momentum acting on them (**F***_n_*—normal contact force, **F***_s_*—tangential contact force, and **N**—normal contact vector) [29].

The forces in contact points are calculated from the constitutive laws (Figure 1). This paper used the simple linear elastic (in compression) law. The equations are as follows:

(6) $$F_{n}=K_{n}uN,$$

(7) $$F_{s}=F_{s,prev}+K_{s}\Delta X_{s},$$

where, **F***_n_* and **F***_s_* are the normal and tangential contact forces, respectively (Figure 2), **N** is a unit normal vector on contact points connecting centres of the elements, **X***_s_* denotes the relative tangential displacement increment and **F***_s,prev_* is the tangential force calculated from the previous time step. Contact stiffnesses (i.e., normal stiffnesses *K_n_* and tangential stiffnesses *K_s_*) can be calculated from the following relations:

(8) $$K_{n}=E_{c}\frac{2R_{A}R_{B}}{R_{A}+R_{B}},\;\;\;K_{s}=v_{c}E_{c}\frac{2R_{A}R_{B}}{R_{A}+R_{B}},$$

where *E_c_* is Young’s modulus of particle contact and *ν_c_* is the ratio between normal and tangential contact stiffness. For the normal (in tension) and tangential force, the limit is imposed:

(9) $$F_{s}^{\max}=CR^{2},\;\;\;F_{n}^{\min}=TR^{2},$$

where *C* corresponds to the cohesive contact stress (maximum shear stress at a pressure equal to zero) [29,31] and *T* to the normal tensile contact stress [29] (*R* is a minimum value of *R_A_* and *R_B_*). The current study’s force values are relatively low (significantly lower than the limit); thus, contact break was not considered. 

Moreover, the Coulomb friction is introduced. The model is described differently, depending on whether the contact is broken or not. The equations for the situation before and after the contact break can be expressed as follows, respectively:

(10) $$\left\|F_{s}\right\|-F_{s}^{\max}-\left\|F_{n}\right\|\tan\mu_{c}\leq 0,$$

(11) $$\left\|F_{s}\right\|-\left\|F_{n}\right\|\tan\mu_{c}\leq 0,$$

where $\mu_{c}$ is the Coulomb inter-particle friction angle.

After force calculations, external forces (e.g., gravity or boundary conditions) may be added. In the next step, the motion of the elements is upgraded using Newton’s second law. The acceleration, velocity, and finally, new particle position are calculated. Then, the first step is repeated. Note that in this paper, no damping is introduced (however, usually, for quasi-static calculations, it is necessary to use it). In the first step, the initial overlapping was calculated (*u*_int_). Next, in every future step, Equation (6) was changed into:

(12) $$F_{n}=K_{n}\left(u-u_{\mathrm{int}}\right)N.$$

So, the overlap can exist in the geometry; however, no initial forces are generated.

## 3. A Methodology of Building a DEM Model for Wave Propagation Problems

**Figure 3:**
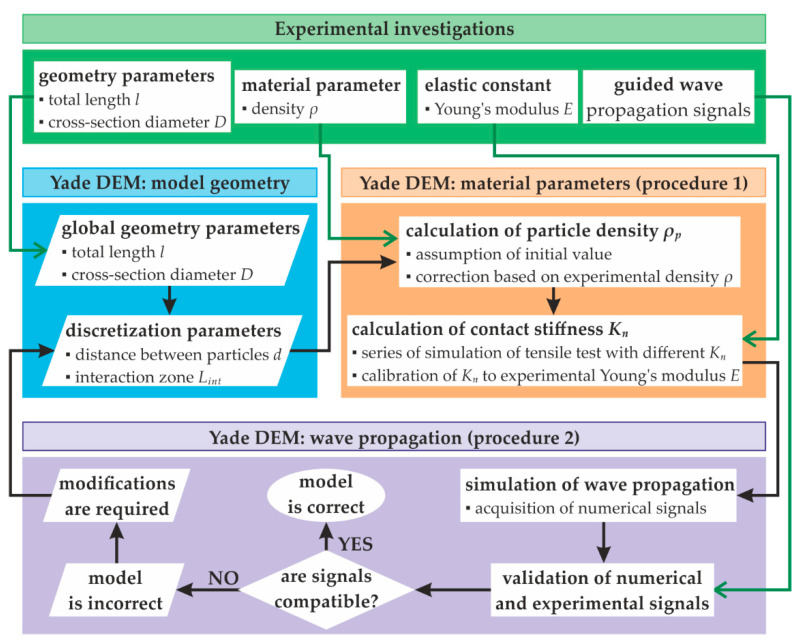
A scheme of building a DEM model of a rod with a circular cross-section for wave propagation using Yade DEM.

The simulation of the guided wave propagation phenomenon using the DEM is much more complex than in the FEM, even in the case of symmetric wave mode propagating in a rod with a circular cross-section. The current research proposes to build an appropriate model based on the calibration with experimental results. The methodology scheme developed using Yade software is presented in Figure 3. At first, it is assumed that the geometry of the rod (length *l*, diameter of the cross-section *D*), the density of the material *ρ*, and Young’s modulus *E* are known (measured on the physical model). Some representative wave propagation signals need to be measured (using the particular excitation signal, e.g., wave packet). The first step of the scheme is the preparation of geometry, reflecting the physical model, including the length and cross-section of the rod. The discretisation with the use of spherical particles with the radius corresponding to the cross-section of the rod is required. The particles are arranged in a single line. An important parameter that must be assumed initially is the distance between the centres of the adjacent particles *d*. Furthermore, the number of adjacent particles with which a specific particle can interact can be set. It is determined by the parameter *L_int_* specifying the area in which the centres of adjacent particles should be included to create an interaction. It is assumed that the specific particle should interact only with the closest neighbours (in general, two, one at each side); thus, the following condition needs to be satisfied:

(13) $$d\leq L_{int}<2d.$$

Having determined the model’s geometry, the calculation of two crucial material parameters, i.e., the density of particles *ρ_p_* and normal contact stiffness *K_n_* needs to be performed using procedure 1 (as presented in Figure 3). First, the initial value of *ρ_p_* is assumed. Based on this value, the total mass of the model is calculated and compared with the actual mass of the physical model. The ratio between these two values is the correction coefficient that is further applied to the initial value of *ρ_p_* to obtain the final particle density. When the value of *ρ_p_* is established, a series of models with different values of *K_n_* is prepared. A simple tensile test simulation is performed for each model to determine the stress-strain relation, also used to calculate Young’s modulus *E_DEM_*. The comparison between the series of *E_DEM_* values and the experimental Young’s modulus *E* allows for choosing the model with the appropriate contact stiffness. The resulting values of *ρ_p_* and *K_n_*, such as the geometry and discretisation previously established, are then incorporated into the second procedure concerning the wave propagation problem. The guided waves are excited and acquired. The signals obtained are compared with the experimental ones to validate the prepared model. The shape of waveforms, such as the wave velocity, can be verified, e.g., by determining the correlation between the two approaches. If the compatibility is satisfactory, the model is considered correct. If not, the model needs modifications, and the algorithm returns to the discretisation. The distance between particles must be changed, and the further steps in procedures 1 and 2 must be repeated. In the present case, if the wave velocity in the numerical signals is too low, the distance between particles needs to be decreased. Inversely, the distance should be increased if the velocity is too high. In general, multiple repetitions of the steps presented can be required.

## 4. Materials and Methods

### 4.1. Object of Research

**Figure 4:**
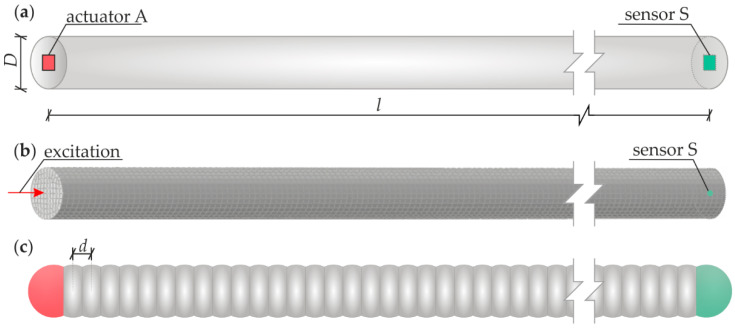
An object of research: (**a**) specimen geometry, (**b**) numerical FEM model, (**c**) numerical DEM model.

The object of the investigation (Figure 4a) was a rod with a circular cross-section (diameter *D* = 10.2 mm) and a length of *l* = 1000 mm. The rod was made of steel, with the following material parameters: mass density *ρ* = 7850.66 kg/m^3^, Young’s modulus *E* = 208.72 GPa (determined in a static tensile test) and Poisson’s ratio *ν* = 0.3.

### 4.2. Experimental Procedure

**Figure 5:**
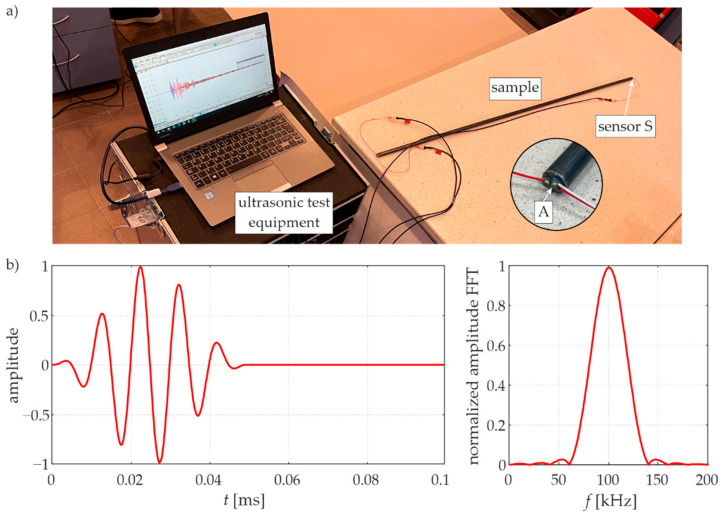
(**a**) Experimental setup. (**b**) Five-cycle sine wave packet with the carrier frequency of 100 kHz (time-domain signal and frequency spectrum).

The experimental measurements of guided waves were carried out using piezoelectric plate transducers Noliac NAC2011 with dimensions of 2 × 2 × 2 mm^3^. One of the transducers acted as an actuator (A), while the second (S) acted as a sensor. The actuator was attached to one end of the rod, while sensor S was attached to the other end, as shown in Figure 5a, so the waves were excited and sensed in the longitudinal direction. The wave packet induced by the actuator was a five-cycle sine function modulated with a Hann window (Figure 5b). The central frequency of the wave packet was set in the frequency range of 50–150 kHz with a step of 10 kHz. Signals were further processed with the Hilbert transform to obtain signal envelopes [32].

### 4.3. Numerical Modelling

#### 4.3.1. Finite Element Method

Numerical analysis was performed in the Abaqus software based on the finite element method. The created 3D model of a bar (Figure 4b) reflected the experimental object (including geometry and mechanical parameters). A linear-elastic, homogeneous, isotropic material model was used. Rayleigh proportional damping was assumed with a mass proportionality coefficient equal to *α* = 2000 1/N, neglecting the influence of stiffness (*β* = 0 m/N). The use of damping allows one to reflect the real wave propagation in the tested object. Boundary conditions were assumed as free edges. The numerical model was made of solid 8-node finite elements with reduced integration (C3D8R). The mesh grid had a size of 1 × 1 × 1 mm^3^. The explicit module was used to calculate the guided waves propagation problem. An algorithm of the central difference method has been applied to integrate the equation of motion. The total calculation time was assumed to be 1.5 ms with the time step equal to 1·10^−7^ s. The wave was excited by applying a concentrated force of a certain amplitude at one end of the rod. The input signal was the same as in the experimental investigations. The results of the analysis were recorded at the point at the opposite end.

#### 4.3.2. Discrete Element Method

Numerical calculations based on the discrete element method were performed in the Yade environment. There are a few significant parameters in wave propagation calculations: the particle density *ρ_p_*, normal contact stiffness *K_n_*, the geometry (initial distance between the spheres *d*) and the coordination number (the number of contacts for every element, determined by the interaction zone *L_int_*). In the first step, samples with a predetermined geometry were prepared. The steel bar was created as a 3D model as one row of spherical elements with a length of *l =* 1000 mm (Figure 4c). The radius of each particle was constant and equal to half the diameter of the real bar (*R =* 5.1 mm). The parameter of the initial distance of the particle *d* has been changed. The interaction zone *L_int_* was chosen so that the specific particle interacted only with its nearest neighbours. For such prepared geometry, the quasi-static tension test was performed (only elastic part, with no breakage). The density parameter was unique for a specified distance to keep the overall mass of the bar in agreement with the real value. The modification of the stiffness coefficient directly affects the global Young’s modulus *E_DEM_*. The prepared model was used for the 1D problem of longitudinal wave propagation. An explicit procedure was used, and the time step equal to *dt =* 5 × 10^−8^ s was adopted (critical time step *dt_cr_ =* 1.3 × 10^−5^ s). The total computation time was 1.5 ms. No additional damping was applied to the model; nevertheless, attenuation was observed in the signals (resulting from the geometrical damping). The disturbance was induced by force applied to the first particle. The signal was recorded at the last particle (at the end of the bar, sensor S).

## 5. Results

A series of calculations were carried out to calibrate the DEM numerical model with the experiment. In the following steps, due to the size of the calculation, only the results for selected particle distances *d* = {0.95*R*, 1.03*R*, 1.05*R*, 1.15*R*} are presented. This set was chosen to show all aspects of the considered issue and the arising difficulties as clearly as possible. The experimental signal was taken as a reference one, to which the numerical signals were matched. The adjustment accuracy was determined qualitatively by visual evaluation of the signals and quantitatively using the Pearson correlation coefficient (PCC), such as the sum of squared errors of the longitudinal wave velocities.

**Figure 6:**
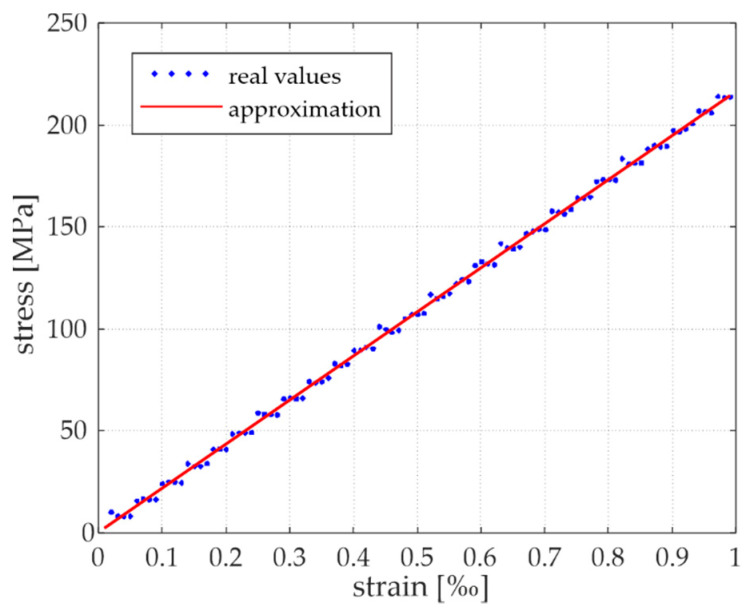
Stress-strain relation for the 1.03*R* model for the determination of *K_n_*.

The first step of the calculation for each different distance was to determine the static parameter (Young’s modulus). The static tensile test simulation was performed for different values of contact stiffness *K_n_* (assumed heuristically). Young’s modulus *E_DEM_* was determined for each calculation by linear approximation of the stress-strain relation obtained from the measurements. The final value of the contact stiffness *K_n_* corresponded to the model with an *E_DEM_* value close to the real value of *E*. Figure 6 presents an example of the stress-strain relationship and its approximation for the model with *d* = 1.03*R*.

**Figure 7:**
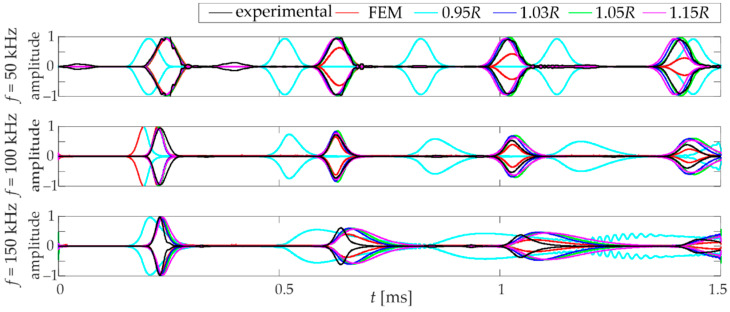
Comparison of signal for selected DEM models (various overlap) and frequencies.

Having determined all the appropriate parameters, the guided wave propagation calculations were carried out. Figure 7 shows the signals of the longitudinal wave collected at the sensor. The signals for four selected distances of particles (*d =* {0.95*R,* 1.03*R,* 1.05*R*, 1.15*R*}) are presented. Excitation frequencies equal to 50 kHz, 100 kHz, and 150 kHz were analysed. Firstly, there is a decrease in velocity with increasing frequency. When considering the models’ signals with different particle distances, it is evident that the *d =* 0.95*R* strongly deviates from the others. The densification of the rod particles implies an increase in the propagation speed of the wave. Moreover, a numerical dispersion is much more pronounced (in comparison to the three other models) at higher frequencies. When comparing the waveforms presented, it can be concluded that the 0.95*R* model is the most unstable.

Correlation calculations were performed using the PCC to determine which model is the most unambiguously compatible with the experiment. Table 1 shows the estimated static parameters for selected models with different particle distances with correlation coefficients. The comparison is intended to show the influence of the agreement distance parameter on the obtained agreement with reference (experimental) measurements and FEM calculations. A strong effect of the distance applied in the DEM model on the obtained correlation can be observed. This confirms previous observations, that the signals received for *d =* {1.03*R*, 1.05*R*, 1.15*R*} are comparable. Their average correlation coefficients (for all particular frequencies) indicate high similarity. However, this requires verification over a wider frequency range.

**Figure 8:**
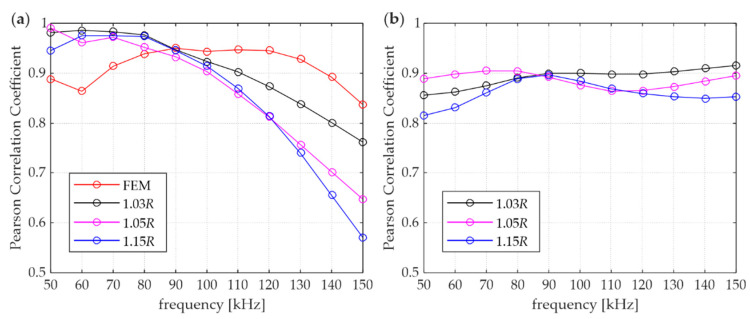
Pearson correlation coefficient in relation to excitation frequency: (**a**) FEM and DEM vs. experimental results, (**b**) DEM vs. FEM results.

Figure 8 presents the PCC variation in relation to the wave frequency. The correlation between numerical (FEM and DEM with different overlaps) and experimental waveforms are shown in Figure 8a. First, it can be observed that there is a rapid decrease in the PCC at higher frequencies. The decrease is related to the presence of an increasing numerical dispersion. A good agreement between the experimental and FEM results can be observed (PCC above 0.85). In the case of DEM results, the PCC is above 0.7 only for the 1.03*R* model; it could be considered a strong correlation [33]. When the experimental vs. DEM correlation curves are considered, a characteristic decrease in the PCC value is observed with an increase in the distance between the particles. Furthermore, it can be seen that in the frequency range of 50–90 kHz, the PCC values of DEM vs. experimental signals are noticeably higher than the PCC values of FEM vs. experimental signals, but above a frequency of 100 kHz FEM model gives a stronger correlation. Figure 8b illustrates a plot of the PCC calculated between the FEM and DEM signal envelopes. A strong agreement between the methods is shown within the analysed frequencies. The PCC for all models ranges between 0.8 and 1.0. This confirms that these two different numerical approaches can give comparable results.

**Figure 9:**
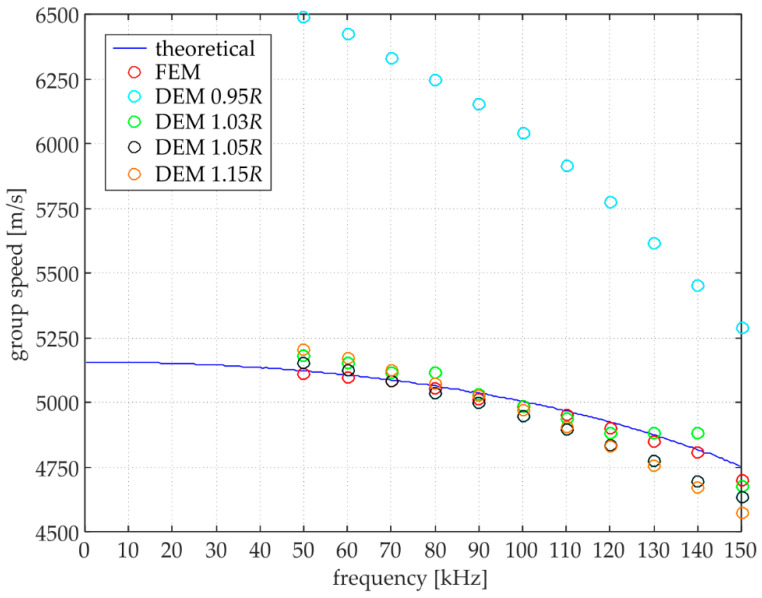
Comparison between dispersion curves for numerical models and Pochhamer-Chree theory.

For wave propagation analysis, it is crucial to recognise the relationship between velocity and frequency. Thus, the characteristic dispersion curves were determined for the steel bar analysed. Theoretical curves were obtained using PCDISP software based on the Pochhammer-Chree theory [34,35]. The numerical FEM/DEM curves were obtained by calculating wave propagation velocity based on two adjacent wave packets using the ‘peak to peak’ method. Figure 9 shows the dispersion curves obtained by different approaches. The DEM curves for four different distances were compared with the theoretical ones. This comparison is intended to highlight the accuracy of the adopted discrete model. First of all, it is worth noting that the obtained curves indicate the dispersive nature of the waves in DEM. Second, it can be pointed out that the group velocity is closely related to the applied particle distance. The shorter the distances between the particle’s centres, the faster the wave propagates. As the distance increases, the group velocity decreases. Additionally, as the frequency increases, the velocity decreases. In the case of *d =* 1.05*R* and *d =* 1.15*R*, the curves that fit the theoretical curve at lower frequencies start to deviate increasingly from the theory as the frequency increases. The dispersion curve obtained for the DEM model at *d =* 1.03*R* was selected to present the greatest agreement with the theory and the numerical FEM result (the group velocity of the numerical FEM is in accordance with the theoretical one at all frequencies). To determine the degree of similarity and confirm the proposed solution’s validity, the residual sum of squares (RSS) was calculated [5]. The results obtained are summarised in Table 2. The RSS values clearly show the agreement of the applied models. Analysing the values for the numerical DEM models, it can be observed that the best agreement (the lowest value of RSS) is shown for the *d =* 1.03*R* model.

**Figure 10:**
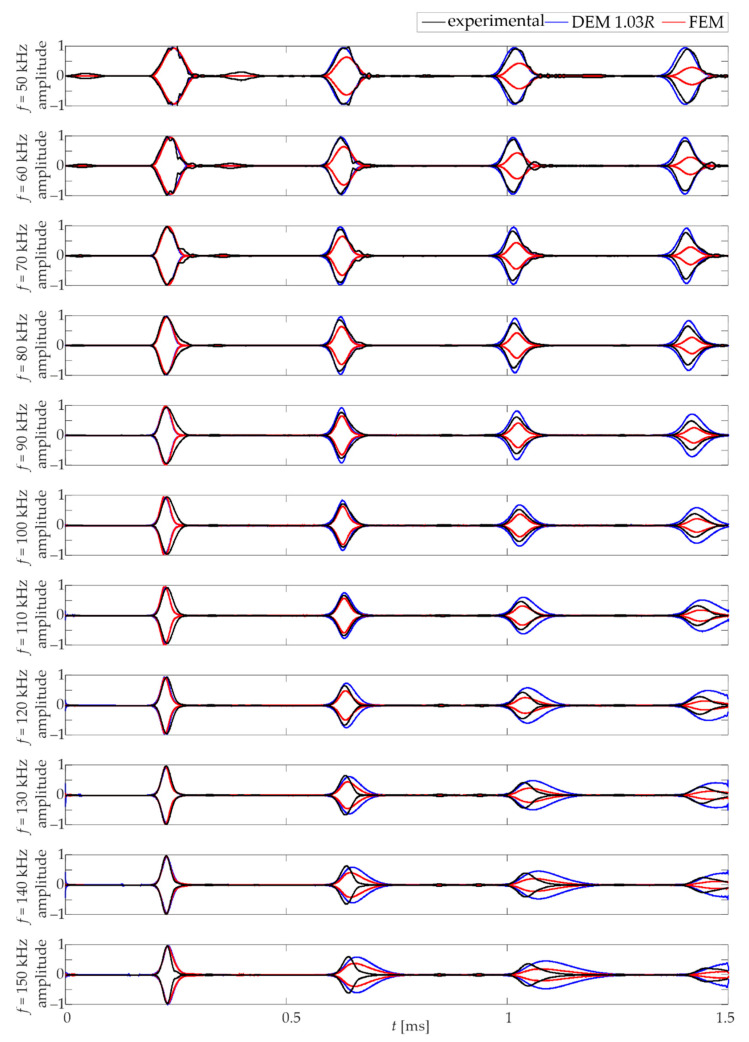
Comparison of wave propagation for experimental and numerical FEM/DEM results.

The wave propagation results for the final DEM model (*d =* 1.03*R*) are shown in Figure 10. The experimental results are compared with the numerical results obtained from FEM and DEM calculations. By analysing the graphs, it is possible to observe the compatibility of the speed of wave propagation and the compatibility of the shape of the wave packets. The first wave packets that appear are compatible with each other. The satisfactory compatibility of guided wave calculations was achieved using the discrete element and finite element methods. As mentioned earlier, the superiority of DEM over FEM can be seen at lower frequencies (50–90 kHz). The packet shapes obtained in the DEM calculations reflect the experimental ones clearly enhanced. A characteristic effect of dispersion is seen in both numerical models at higher frequencies. There is an elongation of the numerical packets, while their beginnings are consistent with the experimental ones.

## 6. Conclusions

The paper describes the modelling of longitudinal wave propagation in a circular steel rod using the discrete element method. An experimental approach was applied to verify the appropriateness of DEM modelling in the Yade open-source code. The finite element calculations performed in Abaqus software supported the performed analyses. Based on the results, the following conclusions could be formulated.

- The Yade platform can be successfully used for experimentally assisted guided wave modelling. The geometric and material parameters need to be determined on a physical model. Moreover, measurements of a certain number of wave propagation signals are required.
- The heuristic process is the appropriate choice of parameters used in DEM models (distance between particles and contact stiffness). The DEM allows one to create several discrete models that fit the experimental results for different sets of parameters. However, further analyses, e.g., dispersion curve calculation, enable selecting the most suitable model.
- Dispersion curves confirmed that guided waves in DEM exhibit a dispersive nature. There is a visible change in group velocity in relation to frequency. Moreover, the wave velocity is closely related to the particle distribution, i.e., the smaller the distances between particles, the higher the group velocity.

In summary, it can be concluded that the discrete element environment can be successfully used for wave propagation analysis. The present work is a beginning consideration for more complex problems, especially to explain the mechanism of propagation and scattering of elastic waves in concrete members at the aggregate level. In the next steps, the calibration of waveforms in concrete and reinforced concrete elements using DEM will be performed. 

## Figures and Tables

**Table 1:** Properties of DEM models.

Model	Number of Particles	Density *ρ_p_* (kg/m^3^)	Normal Contact Stiffness *K_n_* (N/m)	Young’s Modulus *E_DEM_* (GPa)	Correlation with Experiment PCC	Correlation with FEM PCC
0.95*R*	207	5577.17	2.3 × 10^11^	207.30	−0.018	−0.012
1.03*R*	191	6044.37	6.6 × 10^11^	216.30	0.906	0.892
1.05*R*	187	6173.66	6.4 × 10^11^	213.97	0.862	0.886
1.15*R*	171	6751.31	6 × 10^11^	219.37	0.853	0.860

**Table 2:** Residual sum of squares between numerical models and Pochhammer-Chree theory.

Model	RSS
FEM	6056
DEM, 0.95*R*	11,694,645
DEM, 1.03*R*	22,436
DEM, 1.05*R*	61,084

## Data Availability

The data underlying this article will be shared on reasonable request by the corresponding author.
